# TARBP2‐mediated destabilization of Nanog overcomes sorafenib resistance in hepatocellular carcinoma

**DOI:** 10.1002/1878-0261.12449

**Published:** 2019-02-22

**Authors:** Hui‐Huang Lai, Chih‐Wei Li, Chih‐Chen Hong, Hung‐Yu Sun, Ching‐Feng Chiu, Da‐Liang Ou, Pai‐Sheng Chen

**Affiliations:** ^1^ Institute of Basic Medical Sciences College of Medicine National Cheng Kung University Tainan Taiwan; ^2^ Department of Medical Laboratory Science and Biotechnology College of Medicine National Cheng Kung University Tainan Taiwan; ^3^ Institute of Molecular and Genomic Medicine National Health Research Institutes Miaoli Taiwan; ^4^ Department of Biomedical Engineering College of Biology Hunan University Changsha China; ^5^ Graduate Institute of Metabolism and Obesity Sciences College of Nutrition Taipei Medical University Taiwan; ^6^ Graduate Institute of Oncology College of Medicine National Taiwan University Taipei Taiwan

**Keywords:** cancer stem cells, hepatocellular carcinoma, miRNA, Nanog, sorafenib resistance, TARBP2

## Abstract

Hepatocellular carcinoma (HCC) is a lethal human malignancy and a leading cause of cancer‐related death worldwide. Patients with HCC are often diagnosed at an advanced stage, and the prognosis is usually poor. The multikinase inhibitor sorafenib is the first‐line treatment for patients with advanced HCC. However, cases of primary or acquired resistance to sorafenib have gradually increased, leading to a predicament in HCC therapy. Thus, it is critical to investigate the mechanism underlying sorafenib resistance. Transactivation response element RNA‐binding protein 2 (TARBP2) is a multifaceted miRNA biogenesis factor that regulates cancer stem cell (CSC) properties. The tumorigenicity and drug resistance of cancer cells are often enhanced due to the acquisition of CSC features. However, the role of TARBP2 in sorafenib resistance in HCC remains unknown. Our results demonstrate that TARBP2 is significantly downregulated in sorafenib‐resistant HCC cells. The TARBP2 protein was destabilized through autophagic–lysosomal proteolysis, thereby stabilizing the expression of the CSC marker protein Nanog, which facilitates sorafenib resistance in HCC cells. In summary, here we reveal a novel miRNA‐independent role of TARBP2 in regulating sorafenib resistance in HCC cells.

**Abbreviations**
BFA: bafilomycin A1
CHX: cycloheximide
CQ: chloroquine
CSC: cancer stem cell
HCC: hepatocellular carcinoma
miRNA: microRNA
MTT: 3‐(4,5 dimethylthiazol‐2‐yl)‐2,5‐diphenyltetrazolium bromide
NH_4_Cl: ammonium chloride
PKR: dsRNA‐dependent protein kinase
RISC: RNA‐induced slicing complex
SEM: standard error of the mean
SR: sorafenib‐resistant
TARBP2: transactivation response element RNA‐binding protein 2

## 1 Introduction

Liver cancer is the second leading cause of cancer death, accounting for 9% of all types of cancer, and the number of new cancer cases has increased to more than 780 000 annually; thus, liver cancer remains a major health problem worldwide (El‐Serag, 2011; Torre *et al*., 2015). Hepatocellular carcinoma (HCC) is the most common primary malignancy among all primary liver cancers, contributing to ~ 80–90% of cases (El‐Serag, 2011). HCC occurs predominantly in patients with cirrhosis and chronic liver disease, which are chiefly caused by hepatitis B and C viral infections and other risk cofactors, including alcohol consumption, aflatoxin intake, smoking, and metabolic disorders (Balogh *et al*., 2016). Approximately 30–40% of all HCC patients are diagnosed at early stages and receive potentially curative treatments (Llovet *et al*., 2008). Patients in the early stage of HCC are treated with curative treatment options, including liver resection, radiation therapy, and chemotherapy (El‐Serag *et al*., 2008). Chemotherapy drugs, such as floxuridine, cisplatin, and doxorubicin, are the most common treatments, with 5‐year survival rates reaching 75% in HCC patients (Lin *et al*., 2012a; Raza and Sood, 2014). However, HCC is often diagnosed at advanced stages due to the asymptomatic features of early HCC, and the life expectancy of patients with advanced HCC is poor, with a mean survival of 7 months (Giannini *et al*., 2015; Pinter *et al*., 2012). A leading cause of the high mortality rate in HCC patients is the lack of effective therapeutic options, specifically for patients in advanced stages (Colagrande *et al*., 2015). The treatment options for patients with unresectable advanced HCC are extremely limited. Sorafenib is the current recommended standard treatment for advanced HCC (Colagrande *et al*., 2015).

Sorafenib (Nexavar®, BAY 43‐9006, Bayer, Whippany, NJ, USA), an FDA‐approved systemic drug, is the first‐line treatment for improving the survival rate of patients with advanced HCC (Cervello *et al*., 2012; Gauthier and Ho, 2013). As a multikinase inhibitor, sorafenib blocks a broad spectrum of malignant phenotypes, including cell proliferation, tumor angiogenesis, and metastasis of HCC, by repressing the activity of tyrosine kinase receptors to inhibit PI3K/AKT and the Ras/Raf/MAPK pathway (Carlomagno *et al*., 2006; Wellbrock *et al*., 2004; Wilhelm *et al*., 2004). Although sorafenib provides a survival advantage of 2–3 months, the therapeutic effects of sorafenib are temporary, and the response rate in HCC is quite low (Keating and Santoro, 2009). Sorafenib treatment is characterized by high‐level heterogeneity in individual responses, and only ~ 30% of patients benefit from sorafenib treatment. Most patients acquire sorafenib resistance within 6 months, increasing the risk of distant metastasis and cancer recurrence (Chow *et al*., 2013; Keating and Santoro, 2009; Liang *et al*., 2013; Llovet *et al*., 2008; Paez‐Ribes *et al*., 2009). Therefore, it is critical to identify a novel molecular mechanism underlying sorafenib resistance.

Transactivation response element RNA‐binding protein 2 (TARBP2), a well‐known double‐stranded RNA‐binding protein, stabilizes the microRNA (miRNA) biogenesis factor Dicer to induce miRNA maturation, thereby governing the translation of mRNA (Gatignol *et al*., 1991). Additionally, TARBP2 acts as a dsRNA‐dependent protein kinase (PKR) inhibitor to suppress the phosphorylation of eIF2α, thus enhancing cell mitosis and destabilizing transcripts to promote cancer metastasis, exhibiting miRNA‐independent properties in regulating cancer (Garcia *et al*., 2006; Goodarzi *et al*., 2014; Kim *et al*., 2014). Alternatively, the expression of genes related to cancer stem cells (CSCs) in HCC can induce malignant features to promote tumorigenic ability, drug resistance, and metastasis. The CSC markers SOX2, Nanog, and OCT4 are highly expressed in solid tumors and breast, liver, colon, and lung cancers (Dai *et al*., 2013; Karoubi *et al*., 2009; Leis *et al*., 2012; Sun *et al*., 2013), and these markers are associated with drug resistance to tamoxifen, gefitinib, and paclitaxel (Di and Zhao, 2015; Singh and Settleman, 2010; Vinogradov and Wei, 2012). Downregulation of TARBP2 increases Nanog and OCT4 expression and contributes to clonogenicity and tumor growth in Ewing sarcoma family tumors, suggesting that TARBP2 might exhibit potential as a central mediator in regulating the properties of CSC (De Vito *et al*., 2012). Nevertheless, the role of TARBP2 in sorafenib resistance in HCC remains unidentified. Thus, an improved understanding of the underlying molecular mechanism is required to resolve the predicament of current sorafenib therapy. Here, we demonstrate an miRNA‐independent mechanism of TARBP2, in which downregulation of the TARBP2 protein promotes sorafenib resistance in HCC cells through stabilization of Nanog expression.

## 2 Materials and methods

### 2.1 Cell culture

Huh7, Huh7/sorafenib‐resistant (SR), and PLC5 cells were kindly provided by D‐L Ou, Graduate Institute of Oncology, College of Medicine, National Taiwan University, Taipei, Taiwan. PLC5/SR cells were established by long‐term exposure to sorafenib (LC Laboratories, Woburn, MA, USA) at a low dose (5 μm), which was increased to a higher dose (20 μm) over 3 months. HEK‐293T cells were obtained from the American Type Culture Collection. The HEK‐293T, Huh7, PLC5, Huh7/SR, and PLC5/SR cells were grown in Dulbecco's modified Eagle's medium (DMEM)/F12 (HiMedia, MUM, IND). Sorafenib (5 μm) was added to maintain sorafenib resistance in the Huh7/SR and PLC5/SR cells. These cells were supplemented with 10% fetal bovine serum (Gibco, Waltham, MA, USA) and 1% penicillin/streptomycin (GeneDireX, Las Vegas, NV, USA) at 37 °C under 5% CO_2_.

### 2.2 Western blot analysis

The TARBP2‐expressing plasmid from D‐Y Jin was obtained from Addgene (Kok *et al*., 2007). Plasmids expressing Myc‐TARBP2, TARBP2 ΔC4, and Dicer were kindly provided by A. Gatignol (Daniels *et al*., 2009). TARBP2 or TARBP2 ΔC4 was transfected into HCC cells for 48 h using jetPRIME (Polyplus‐transfection, New York, NY, USA and HyFect™ DNA transfection reagent (Leadgene Biomedical, Tainan, Taiwan)). To harvest total proteins, the cells were washed with PBS buffer and lysed in RIPA buffer [Tris‐base (50 mm), NaCl (150 mm), NP‐40 (10%), Na_3_VO_4_ (200 mm), EDTA (100 mm), sodium deoxycholate (0.1%), SDS (1%)]. The cell lysates were sonicated using an ultrasonic processor, and the supernatant was collected after centrifugation at 18 000 ***g*** for 30 min at 4 °C. An equal quantity of protein was resuspended in gel sample buffer and was separated via SDS/PAGE. The proteins separated in the SDS/PAGE were transferred to a polyvinylidene difluoride membrane at 400 mA for 2 h. The membrane was blocked with TBST buffer (0.02 m Tris‐base, 0.15 m NaCl, 5 mL Tween 20, pH 7.5) containing 5% nonfat milk for 1 h at room temperature. After blocking, the membrane was incubated with a specific primary antibody overnight at 4 °C. After washing with TBST buffer, the membrane was hybridized with a horseradish peroxidase‐conjugated secondary antibody for 1 h at room temperature. The membrane was then washed with TBST buffer. Protein expression was visualized using enhanced chemiluminescence (PerkinElmer, Waltham, MA, USA). The blots were exposed to autoradiography film to obtain the results.

### 2.3 Isolation of RNA and quantitative real‐time PCR

Total RNA was isolated using the TRIzol reagent (Invitrogen, Carlsbad, CA, USA) according to the manufacturer's protocol. Total mRNA (200 ng) was reverse‐transcribed into cDNA using reverse transcriptase, random primers, dNTPs, and an RNase inhibitor. The parameters for reverse transcription were as follows: 25 °C for 10 min, 42 °C for 45 min, and 70 °C for 15 min. The cDNA was amplified using SYBR™ Green Master Mix (Invitrogen) and gene‐specific primers. The amplified replication signal was detected using the (Applied Biosystems, Waltham, MA, USA) Step One real‐time PCR system according to the manufacturer's protocols. The PCR cycling parameters were as follows: 95 °C for 3 min and 40 cycles of 95 °C for 15 s, 60 °C for 1 min and 75  °C for 15 s. The primers used to detect the specific sequences were as follows: TARBP2 (F: 5′‐GGG CTC TTG GGT TCT GTA GT‐3′; R: 5′‐GTT TGT AAT ACC GTC CCG CC‐3′), Nanog (F: 5′‐ATA GCA ATG GTG TGA CGC AG‐3′;R: 5′‐ACC AGG TCT GAG TGT TCC AG‐3′), GAPDH (F: 5′‐ACC CAC TCC TCC ACC TTT GAC‐3′; R: 5′‐TCC ACC ACC CTG TTG CTG TAC‐3′). GAPDH was used as an endogenous control to normalize TARBP2 and Nanog expression.

### 2.4 Cell viability analysis

Cell viability was determined using the 3‐(4,5 dimethylthiazol‐2‐yl)‐2,5‐diphenyltetrazolium bromide (MTT) assay. The cells were seeded in triplicate at a density of 3500 cells per well in 96‐well plates. After 24 h, the cells were treated with the indicated concentrations of sorafenib for 48 h. The cells were then treated with MTT solution (5 mg·mL^−1^) for 2 h. Next, the medium was removed, and 100 μL of DMSO was added to each well to dissolve the insoluble purple formazan product. The absorbance of the colored solution was measured at 570 nm using a spectrophotometer. All experiments were performed in triplicate.

### 2.5 shRNA‐packaged lentivirus knockdown

pCMVΔR8.91, pMD.G, TARBP2, Nanog, and GFP short hairpin‐constructed plasmids were purchased from the National RNAi Core Facility Platform located at the Institute of Molecular Biology/Genomic Research Center, Academia Sinica. For lentivirus production, HEK‐293T cells were cotransfected with a constructed short hairpin‐carrying plasmid (1 μg), pCMVΔR8.91 (5 μg), and pMD.G (5 μg). After transfection for 24 h, the supernatant was collected and filtered through a 0.45‐μm filter (Millipore, Billerica, MA, USA). HCC cells were seeded in 10‐cm dishes containing DMEM/F12. The lentivirus and polybrene (1 μg·mL^−1^) were added to the cells, followed by incubation for 48 h at 37 °C under 5% CO_2_. The medium was replaced with fresh medium supplemented with 1 μg·mL^−1^ puromycin to select stable clones. After 48 h of selection, the culture medium was removed and replaced with fresh medium containing 0.5 μg·mL^−1^ puromycin to maintain the gene knockdown of stable clones.

### 2.6 Sphere formation

Cells were trypsinized and suspended to generate single cells, for seeding at a density of 1000 cells per well in nonadherent plates in serum‐free DMEM/F12 medium, with epidermal growth factor (50 ng·mL^−1^), basic fibroblast growth factor (50 ng·mL^−1^; R&D Systems, Minneapolis, MN, USA), and 1× B27 supplement (Invitrogen) for 14 days. Quantification of sphere formation was performed by directly counting the number of spheres per well in plates.

### 2.7 HCC xenograft model of acquired resistance to sorafenib

The protocol for the xenograft experiments was approved by the Institutional Animal Care and Use Committee of the College of Medicine, National Taiwan University. All animal experiments were performed according to the criteria outlined in the Guide for the Care and Use of Laboratory Animals prepared by the National Academy of Sciences and published by the National Institutes of Health. Male BALB/c athymic (nu^+^/nu^+^) mice were inoculated subcutaneously with Huh7 cells. When tumors reached a 100 mm^3^ volume, mice were treated with sorafenib or placebo. Sorafenib (10 mg·kg^−1^·day^−1^) was administered daily via gavage. Tumor volume and body weight were recorded every 7 days. At the end of sorafenib treatment, the tumor samples were grouped into two groups: SR (tumor volume > 1000 mm^3^) and sorafenib‐sensitive (SS; tumor volume < 1000 mm^3^) tumors. The tumor samples were collected for western blotting (Hsu *et al*., 2016).

### 2.8 Bioinformatics analysis

Oncomine was used for the analysis and visualization of the TGCA liver cancer datasets (http://www.oncomine.org) (Rhodes *et al*., 2004). SurvExpress is a biomarker validation tool and database for the integration of cancer gene expression and clinical outcome data (http://bioinformatica.mty.itesm.mx/SurvExpress) (Aguirre‐Gamboa *et al*., 2013). PRECOG is a bioinformatics tool for analyzing the associations between genomic profiles and cancer outcomes (http://precog.stanford.edu/) (Fernandez‐Ricaud *et al*., 2016).

### 2.9 Statistical analysis

All data are expressed as the mean ± standard error of the mean (SEM) from at least three individual experiments. One‐way ANOVA was used to analyze statistically significant differences among multiple groups. Two‐way ANOVA was used to analyze multiple groups with two categorical variables. All analyses were performed using prism 6.0 software (GraphPad Software Inc., San Diego, CA, USA). *P* values of < 0.05 were considered statistically significant. *P < 0.05, **P < 0.01, or ***P < 0.005.

## 3 Results

### 3.1 TARBP2 downregulation correlates with a poor outcome in patients with HCC and enhances sorafenib resistance in HCC cells

**Figure 1:**
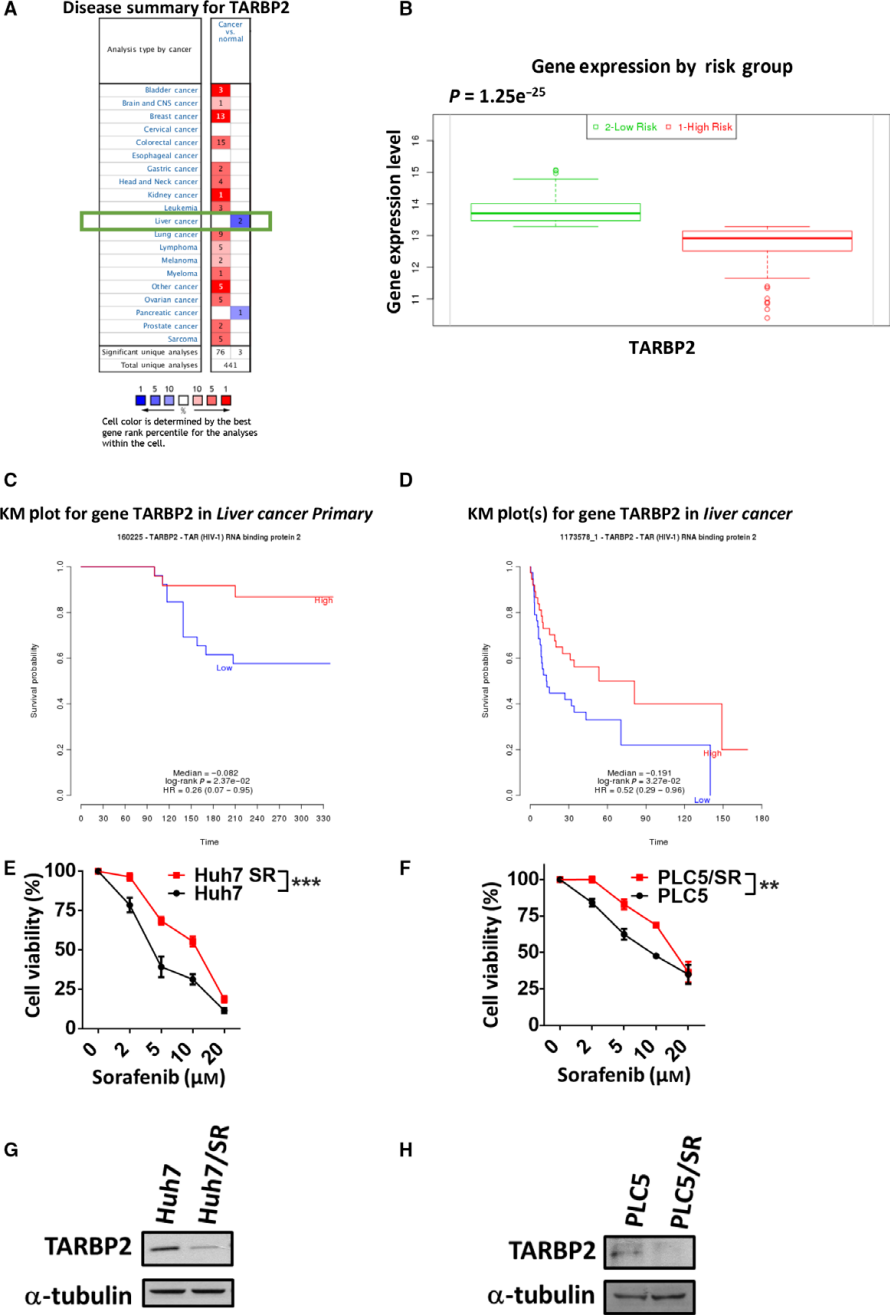
Downregulation of TARBP2 correlates with a poor outcome of patients with HCC and facilitates SR in HCC cells. (A) *TARBP2* mRNA expression data were collected from the Oncomine database with thresholds of a *P* value ≤ 0.01 and gene rank ≤ 10%. The numbers in the colored cells represent the number of analyses. The red cells indicate increased *TARBP2* mRNA expression in tumor tissues; the blue cells indicate reduced *TARBP2* mRNA expression in tumor tissues. (B) The prognostic index of TARBP2 in 162 liver cancer patients was analyzed from the SurvExpress database and categorized into low‐ and high‐risk groups (*x*‐axis). The expression of TARBP2 is presented along the *y*‐axis. (C, D) Kaplan–Meier curves were generated from the PRECOG database. The data were collected from groups of 50 (GSE364; C) and 91 (GSE1898; D) liver cancer patients. (E, F) Expression of TARBP2 in paired HCC cell lines. Huh7 and Huh7/SR (E) or PLC5 and PLC5/SR (F) cells were treated with the indicated concentrations of sorafenib for 48 h. Cell viability was measured using the MTT assay. Data are presented as mean ± SEM, with at least *n* = 3 per group. Multigroup comparisons were analyzed by two‐way ANOVA with Tukey's *post hoc* test. *P* values < 0.05 were considered statistically significant. **P <* *0.01; or ***P <* *0.005. (G and H) The expression of TARBP2 in Huh7 (G) and PLC5 (H) cells was determined via western blot analysis.

To determine the significance of TARBP2 in the clinical outcome of patients with HCC, TARBP2 was validated via bioinformatics database analysis. Data on *TARBP2* mRNA expression in normal and tumor tissues were collected from 441 unique analyses in the Oncomine database (Rhodes *et al*., 2004). TARBP2 expression was observed to be significantly elevated in most cancer types. In particular, TARBP2 expression was relatively decreased in liver cancer and pancreatic cancer, suggesting that TARBP2 levels were suppressed in liver and pancreatic tumors (Fig. 1A). The prognostic index of TARBP2 in liver cancer patients was analyzed using the SurvExpress database (Aguirre‐Gamboa *et al*., 2013), and the patients were categorized into low‐ and high‐risk groups based on their survival time and status. TARBP2 was downregulated in the high‐risk group, which was associated with a poor prognosis in patients with HCC (Fig. 1B; *P *= 1.25e‐25). Additionally, to correlate TARBP2 expression with the survival of HCC patients, we mined the PRECOG database to collect survival rates from groups of 50 (Fig. 1C; HR = 0.26; 95% CI: 0.07–0.95, *P *= 2.37e‐02) and 91 patients (Fig. 1D; HR = 0.52; 95% CI: 0.29–0.96, *P *= 3.27e‐02) with liver cancer for Kaplan–Meier survival analysis (Fernandez‐Ricaud *et al*., 2016). These clinical results showed that downregulation of TARBP2 was correlated with a poor prognosis and survival rate of patients with HCC. Based on clinical evidence revealing the significance of TARBP2 downregulation in HCC patients, we further investigated the molecular mechanism underlying SR in HCC cells. We first established two SR HCC cell lines, from Huh7 and PLC5 parental cells, via repeated long‐term exposure of the cancer cells to sorafenib at increasing dose concentrations (5–20 μm) for 3 months. To examine the level of sorafenib resistance in the HCC cell lines, the cells were treated with sorafenib in a dose‐dependent manner for 48 h, and cell viability was measured using the MTT assay. The inhibitory concentrations (IC_50_) of sorafenib in Huh7 and Huh7/SR cells were 4.72 ± 0.67 and 9.66 ± 0.99 μm, respectively (Fig. 1E). The IC_50_ values of sorafenib in PLC5 and PLC5/SR cells were 9.63 ± 0.98 and 14.84 ± 1.17 μm, respectively (Fig. 1F). These results indicated that the HCC cells were stably resistant to sorafenib, and these paired cell lines were used for further investigations. To determine the role of TARBP2 in sorafenib resistance in HCC cells, TARBP2 protein expression was analyzed using western blotting. The TARBP2 protein was significantly downregulated in SR HCC cells (Fig. 1G,H). TARBP2 is suppressed in HCC/SR cells, suggesting that downregulation of TARBP2 enhances sorafenib resistance in HCC cells.

### 3.2 TARBP2 suppressed sorafenib resistance of HCC cells is miRNA‐independent

**Figure 2:**
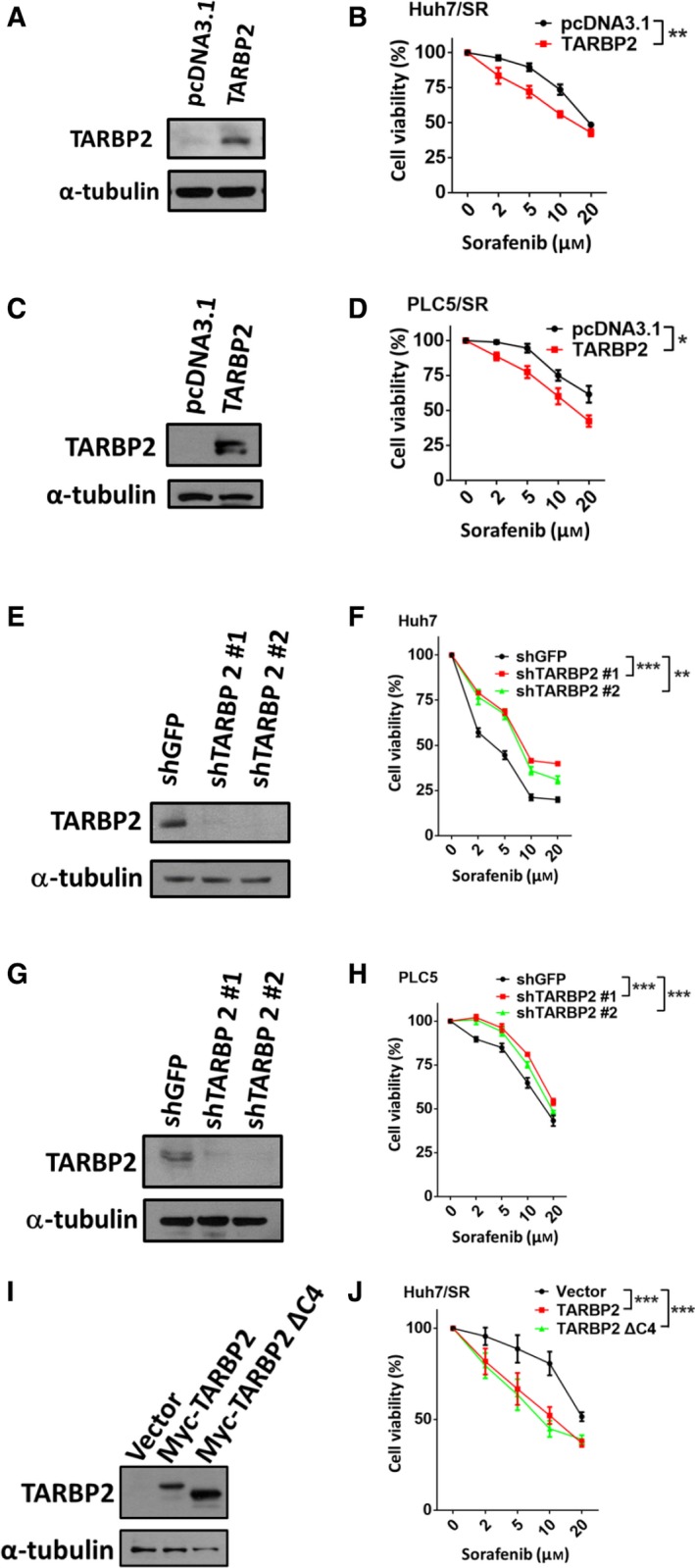
Downregulation of TARBP2 enhances SR in HCC cells. (A–H) Effect of TARBP2 expression on SR in HCC cells. TARBP2 was overexpressed in Huh7/SR cells (A) and PLC5/SR cells (C) for 48 h. TARBP2 was knocked down in Huh7 (E) and PLC5 (G) cells. TARBP2 protein expression was determined via western blot analysis. Cell viability was measured using the MTT assay (B, D, F, and H). (I, J) Effect of TARBP2 ΔC4 expression on SR in Huh7/SR cells. TARBP2 and truncated C4 TARBP2 protein expression was determined via western blot analysis (I). Cell viability was measured via the MTT assay (J). Data are presented as mean ± SEM, with at least *n* = 3 per group. Multigroup comparisons were analyzed by two‐way ANOVA with Tukey's *post hoc* test. *P* values < 0.05 were considered statistically significant. *P <* *0.05; **P <* *0.01; or ***P <* *0.005.

To determine the function of TARBP2 in sorafenib resistance in HCC cells, TARBP2‐overexpressing HCC cells were treated with sorafenib at the indicated concentrations (0, 2, 5, 10, and 20 μm) for 48 h (Fig. 2A,B). The MTT assay demonstrated that TARBP2 significantly sensitized the Huh7/SR cells to sorafenib treatment (Fig. 2B). Accordingly, overexpression of TARBP2 decreased the level of sorafenib resistance in PLC5/SR cells (Fig. 2C,D), suggesting that TARBP2 functions as a tumor suppressor by sensitizing = HCC cells to sorafenib treatment. To further confirm the function of TARBP2 in the parental HCC cells, TARBP2 was knocked down using two specific *TARBP2*‐CDS‐targeting short hairpin RNAs in Huh7 and PLC5 cells, which expressed higher levels of TARBP2. The TARBP2‐knockdown HCC cells were treated with the indicated concentrations (0, 2, 5, 10, and 20 μm) of sorafenib for 48 h (Fig. 2E,F). Knockdown of TARBP2 significantly enhanced sorafenib resistance in Huh7 cells (Fig. 2F). Inhibition of TARBP2 expression promoted sorafenib resistance in PLC5 cells (Fig. 2G,H), indicating that downregulation of TARBP2 facilitates sorafenib resistance in HCC cells. TARBP2 is an essential biogenesis factor in the RNA‐induced slicing complex (RISC) for miRNA biogenesis (Gatignol *et al*., 1991). C4‐domain‐truncated TARBP2 was used to disrupt the binding between TARBP2 and Dicer and block miRNA biogenesis (Daniels *et al*., 2009) to investigate whether the TARBP2‐enhanced sensitivity of HCC cells to sorafenib treatment is miRNA‐dependent (Fig. 2I,J). Cell viability was decreased in Huh7/SR cells expressing wild‐type TARBP2 and those expressing TARBP2 ΔC4 (Fig. 2J), suggesting that TARBP2‐mediated sensitization of HCC cells to sorafenib treatment is miRNA‐independent.

### 3.3 TARBP2 protein is destabilized in sorafenib‐resistant HCC cells

**Figure 3:**
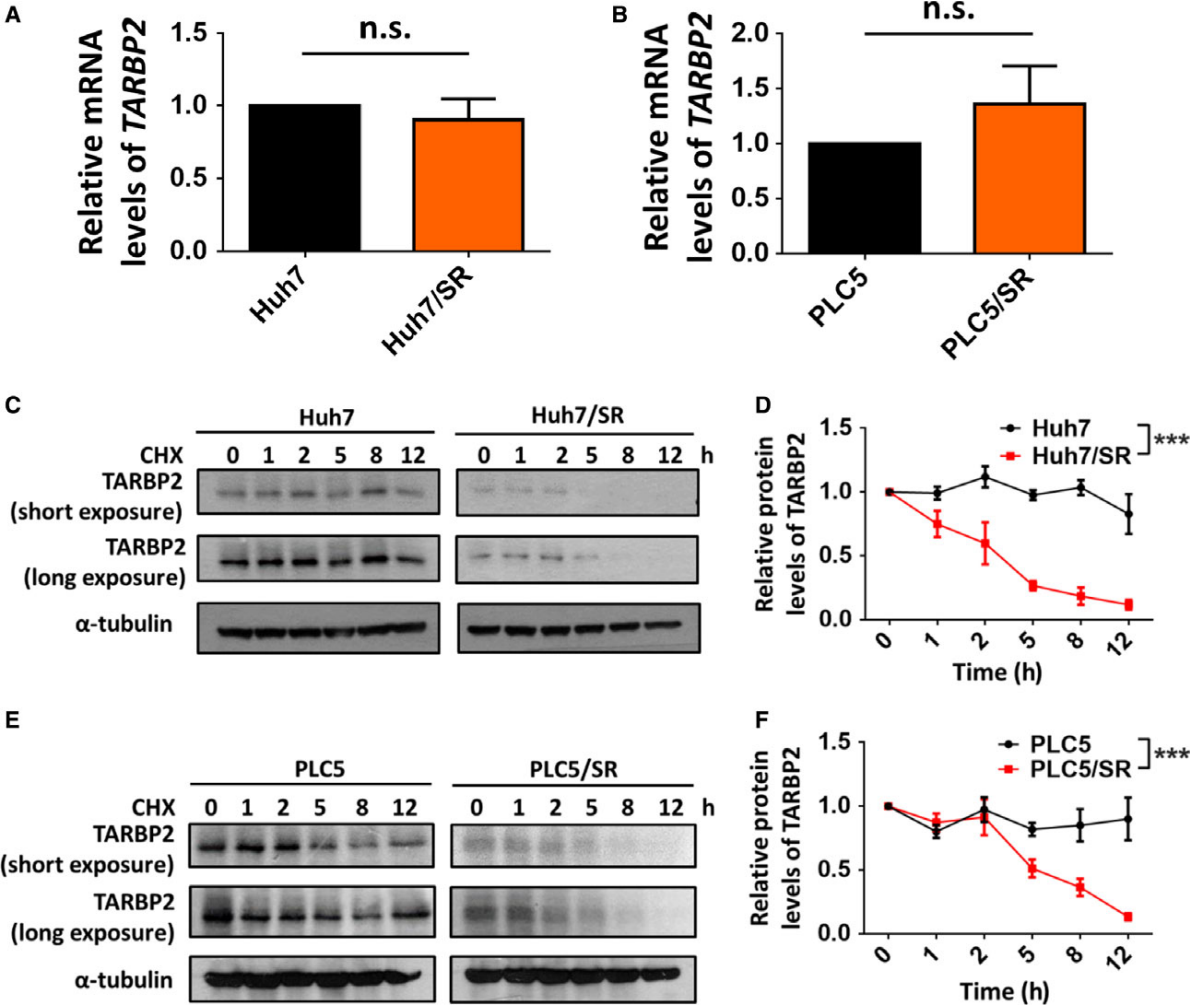
TARBP2 protein stability is decreased in HCC/SR cells. (A, B) The expression of *TARBP2* mRNA was determined via real‐time PCR in paired Huh7 (A) or PLC5 (B) cell lines. Multigroup comparisons were analyzed by one‐way ANOVA with Tukey's *post hoc* test. (C–F) The TARBP2 protein was destabilized in HCC/SR cells. Huh7 and Huh7/SR (C) or PLC5 and PLC5/SR (E) cells were treated with CHX (100 μg·mL^−1^) for the indicated time periods. The relative quantity of the depicted proteins was analyzed through three independent experiments (D, F). Data are presented as mean ± SEM, with at least *n* = 3 per group. Multigroup comparisons were analyzed by two‐way ANOVA with Tukey's *post hoc* test. *P* values < 0.05 were considered statistically significant. ***P <* *0.005.

After confirming that the TARBP2 protein is suppressed in HCC/SR cells, we next determined whether TARBP2 was downregulated via transcriptional regulation. Quantitative RT/PCR analysis demonstrated that *TARBP2* mRNA levels remained unchanged in both Huh7/SR and PLC5/SR cells (Fig. 3A,B), indicating transcription‐independent regulation of the downregulation of TARBP2 in HCC/SR cells. To further evaluate whether downregulation of the TARBP2 protein occurred through translational or post‐translational regulation, the stability of the protein in the two pairs of HCC cell lines was determined via treatment with the protein synthesis inhibitor cycloheximide (CHX). Huh7 and Huh7/SR cells were treated with CHX for the indicated time periods (Fig. 3C), and the data showed that TARBP2 protein expression was stably maintained for 8 h but was dramatically reduced in Huh7/SR cells starting at 2 h after treatment with CHX (Fig. 3D). Similar effects were observed in the paired PLC5 cell lines (Fig. 3E,F), demonstrating that TARBP2 is downregulated through destabilization of the TARBP2 protein in HCC/SR cells.

### 3.4 The TARBP2 protein is suppressed though autophagic–lysosomal proteolysis in sorafenib‐resistant HCC cells

**Figure 4:**
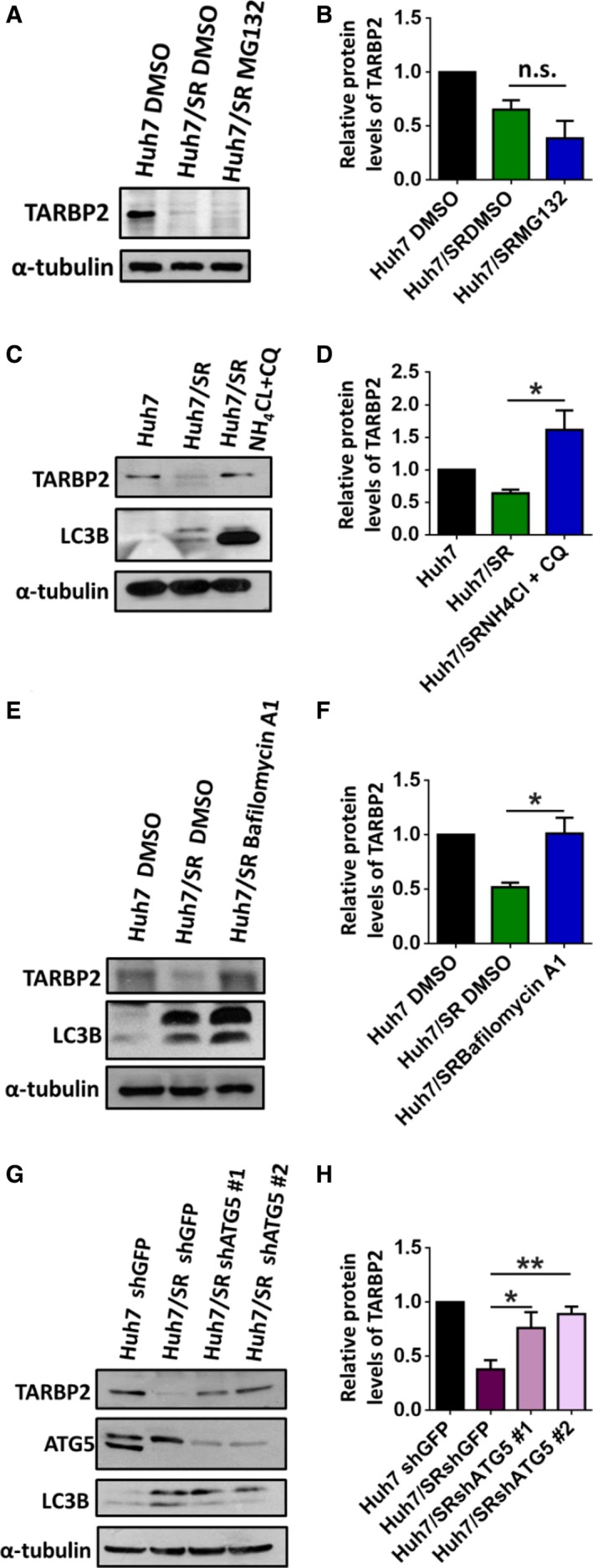
The TARBP2 protein is destabilized through the autophagic–lysosomal pathway. Effects of the proteolytic pathways on TARBP2 downregulation in HCC/SR cells. (A, B) Huh7/SR cells were treated with MG132 (5 μm) for 24 h. (C, D) Huh7/SR cells were treated with NH_4_Cl (10 mm) and CQ (200 μm) for 48 h. (E, F) Huh7/SR cells were treated with BFA (100 nm) for 48 h. (G, H) ATG5 was knocked down in the Huh7/SR cells. The TARBP2, ATG5, and LC3B proteins were quantified via western blot analysis. Data are presented as the mean ± SEM from at least three independent experiments. Multigroup comparisons were analyzed by one‐way ANOVA with Tukey's *post hoc* test. *P* values < 0.05 were considered statistically significant. *P <* *0.05; **P <* *0.01.

In eukaryotic cells, the ubiquitin‐proteasome and lysosome proteolytic pathways are two major pathways of protein degradation and are critical in regulating cancer progression (Glickman and Ciechanover, 2002; Mizushima, 2007). Therefore, we investigated whether the TARBP2 protein is degraded through the proteasome pathway in HCC/SR cells. Huh7/SR cells were treated with MG132 to inhibit proteasome activity. However, inhibition of proteasome‐mediated protein degradation did not prevent TARBP2 downregulation in Huh7/SR cells, suggesting that the degradation of the TARBP2 protein in HCC/SR cells is proteasome independent (Fig. 4A,B). Next, we determined whether the TARBP2 protein was degraded through the lysosomal pathway. Huh7/SR cells were treated with the lysosome inhibitors ammonium chloride (NH_4_Cl) and chloroquine (CQ) to inhibit the activity of lysosomal enzymes through neutralizing the lysosomal pH (Choi, 2012; Hart and Young, 1991). The results demonstrated that TARBP2 protein expression was restored through treatment with NH_4_Cl and CQ in Huh7/SR cells (Fig. 4C,D), indicating that TARBP2 is downregulated through lysosome‐mediated proteolysis. This observation prompted us to further investigate whether autophagy is involved in lysosome‐mediated TARBP2 protein degradation. Autophagy is an intracellular, bulk degradation process that delivers cytoplasmic components to the lysosomes for protein degradation via autophagosomes (Choi, 2012; Mizushima, 2007). To investigate whether the TARBP2 protein is degraded through the autophagic–lysosomal pathway, Huh7/SR cells were treated with bafilomycin A1 (BFA) to inhibit fusion between autophagosomes and lysosomes (Yamamoto *et al*., 1998). TARBP2 protein levels were reconstituted through BFA treatment in Huh7/SR cells (Fig. 4E,F). An increase in LC3B‐II/LC3B‐I conversion indicated the accumulation of autophagosomes. These data suggested that the degradation of TARBP2 occurs via the autophagic–lysosomal pathway in HCC/SR cells. ATG5 induces the formation of a torus‐shaped structure through expanding phagophores for autophagosome formation (Jung *et al*., 2016; Klionsky *et al*., 2003). To further clarify whether the autophagic–lysosomal pathway contributes to TARBP2 protein degradation, ATG5 was knocked down in Huh7/SR cells to inhibit autophagosome biogenesis. Consistent results indicated that the TARBP2 protein level was restored in ATG5‐knockdown Huh7/SR cells (Fig. 4G,H). These results demonstrated that TARBP2 protein degradation occurs through autophagic–lysosomal proteolysis in HCC/SR cells.

### 3.5 TARBP2 reduces the sorafenib resistance of HCC through downregulation of the Nanog protein

**Figure 5:**
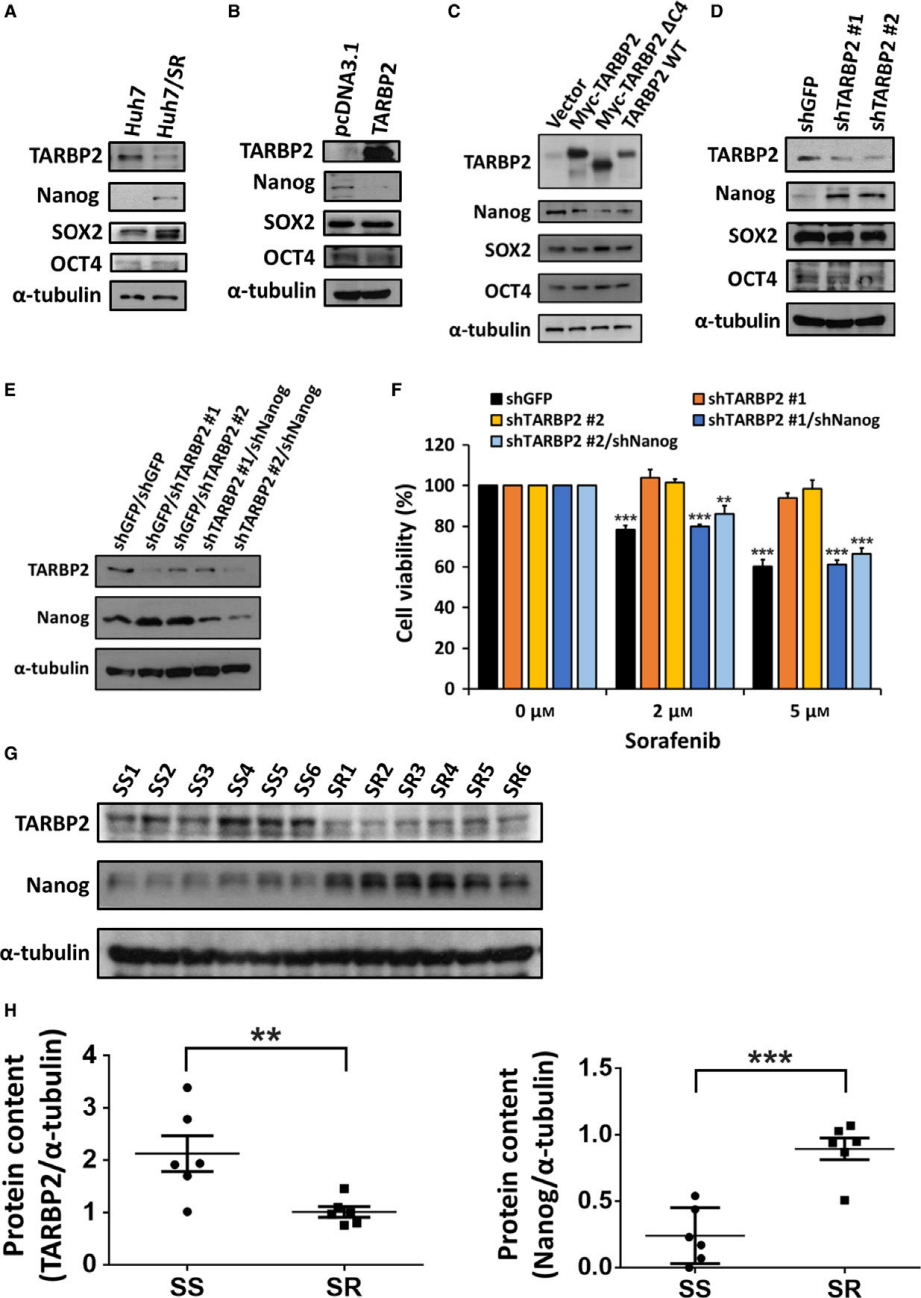
Downregulation of TARBP2 increases Nanog protein levels to promote SR. (A–D) The protein expression of Nanog, SOX2, and OCT4 in paired HCC cells was analyzed via western blot analysis (A). TARBP2 (B) or TARBP2 ΔC4 (C) was overexpressed in Huh7/SR cells for 48 h. TARBP2 was knocked down in Huh7 cells (D). TARBP2 and CSC markers were quantified via western blot analysis. (E, F) Effect of TARBP‐mediated Nanog downregulation on SR. TARBP2 and Nanog were co‐knocked down in Huh7 cells. TARBP2 and Nanog protein levels were determined via western blot analysis (E). The stable clones were treated with the indicated concentrations of sorafenib for 48 h. Cell viability was measured using the MTT assay (F). Data are presented as mean ± SEM, with at least *n* = 3 per group. (G, H) The protein lysates were homogenized from SS and SR tumor tissues (*n* = 6, G). TARBP2 and Nanog expression were analyzed by western blot analysis. TARBP2 and Nanog expression were normalized to α‐tubulin to quantify the protein content by using imagej software (National Institutes of Health, Bethesda, MD, USA) (H). Multigroup comparisons were analyzed by one‐way ANOVA with Tukey's *post hoc* test. *P* values < 0.05 were considered statistically significant. **P <* *0.01; ***P <* *0.005.

Because TARBP2 protein downregulation occurred through the autophagic–lysosomal pathway and induced sorafenib resistance in HCC cells, we further investigated the components downstream of TARBP2 in sorafenib resistance in SR cells. CSCs, known as tumor‐initiating cells, exhibit a high ability to enhance tumorigenesis, metastasis, chemotherapy, and radiation resistance (Magee *et al*., 2012). The CSC markers SOX2, OCT4, and Nanog have been demonstrated to promote the resistance of cancer to drugs including sorafenib, tamoxifen, cisplatin, and paclitaxel (Di and Zhao, 2015; Shan *et al*., 2012; Singh and Settleman, 2010; Vinogradov and Wei, 2012). As previously described, TARBP2 inhibits CSC marker expression in Ewing sarcoma (De Vito *et al*., 2012). However, the role of TARBP2 in regulating CSC markers in HCC cells remains unclear. Thus, we examined the protein levels of Nanog, SOX2, and OCT4 in the paired HCC cells. Nanog protein expression was significantly increased in Huh7/SR cells (Fig. 5A). Next, we confirmed Nanog protein expression by manipulating TARBP2 expression in the paired Huh7 cell lines. Nanog protein expression was suppressed in TARBP2‐overexpressing Huh7/SR cells (Fig. 5B). To examine whether TARBP2‐mediated Nanog suppression is miRNA‐dependent, TARBP2 ΔC4 was overexpressed in Huh7/SR cells. Nanog remained suppressed in the TARBP2 ΔC4‐overexpressing Huh7/SR cells, suggesting that TARBP2‐mediated Nanog suppression is miRNA‐independent (Fig. 5C). Additionally, knockdown of TARBP2 increased the expression of the Nanog protein in the Huh7 cells (Fig. 5D). Supporting previous results, SOX2 and OCT4 expression exhibited no obvious difference in TARBP2‐overexpressing, TARBP2‐ΔC4‐overexpressing or TARBP2‐knockdown Huh7/SR and Huh7 cells (Fig. 5B–D). These results indicated that TARBP2‐mediated Nanog protein inhibition is miRNA independent. To further investigate the biological consequences of TARBP2‐induced Nanog suppression in HCC cells, TARBP2 and Nanog were co‐knocked down in Huh7 cells through sorafenib treatment to detect the level of sorafenib resistance (Fig. 5E,F). The MTT assay demonstrated that the knockdown of TARBP2 enhanced sorafenib resistance, whereas co‐knockdown of Nanog resensitized the Huh7 cells to sorafenib treatment (Fig. 5F). These results suggested that downregulation of TARBP2 enhances sorafenib resistance through stabilization of the Nanog protein in HCC/SR cells. To evaluate this mechanism *in vivo*, we generated an HCC xenograft model of acquired resistance to sorafenib. Male BALB/c athymic mice were subcutaneously injected with Huh7 cells. After tumor growth reached a volume of 100 mm^3^, the mice were treated with placebo or sorafenib (10 mg·kg^−1^·day^−1^) via gavage. At the end of sorafenib treatment, the tumor samples were classified into SR (tumor volume > 1000 mm^3^) and SS (tumor volume < 1000 mm^3^) groups. The individual tumor samples were collected for western blot analysis. The *in vivo* evidence showed that TARBP2 was significantly decreased in SR tumor tissues, whereas Nanog expression was upregulated (Fig. 5G,H). Nanog is a transcription factor that is activated in embryonic stem (ES) cells and has been demonstrated to play a role in the chemoresistance of liver CSCs (Chiba *et al*., 2006; Lee *et al*., 2011). Genetic changes regulate the cellular stemness of liver CSCs, where specific cell surface markers and functional markers are activated to maintain the features of these cells, including CD24, CD44, CD133, EpCAM, and ALDH1 (Ma *et al*., 2008; Yamashita and Wang, 2013; Yamashita *et al*., 2009). Having confirmed the role of TARBP2 in regulating Nanog expression, we next investigated whether TARBP2 regulated CSC marker expression. CD24, CD44, CD133, EpCAM, and ALDH1 were detected in the paired Huh7 cell lines, and we found that CD44, CD133, EpCAM, and ALDH1 were significantly increased in Huh7/SR cells, whereas these effects were reduced by restoration of TARBP2 (Fig. S1A). We consistently found that these markers were enhanced by the knockdown of TARBP2 in Huh7 cells (Fig. S1B). These results demonstrated that TARBP2 reduces CSC features. We further investigated the sphere formation of Huh7 cells as a representation of their CSC phenotypes (Visvader and Lindeman, 2008). The sphere number was increased in Huh7/SR cells, whereas sphere formation was abolished by the restoration of TARBP2 (Fig. S1C). An increased sphere‐forming capacity was observed in TARBP2‐knockdown Huh7 cells (Fig. S1D), supportively indicating that TARBP2 reduces CSC properties in HCC cells. These *in vitro* results suggested that downregulation of TARBP2 inhibits sorafenib resistance through stabilizing Nanog and maintaining CSC functions.

### 3.6 TARBP2 expression destabilizes Nanog protein

**Figure 6:**
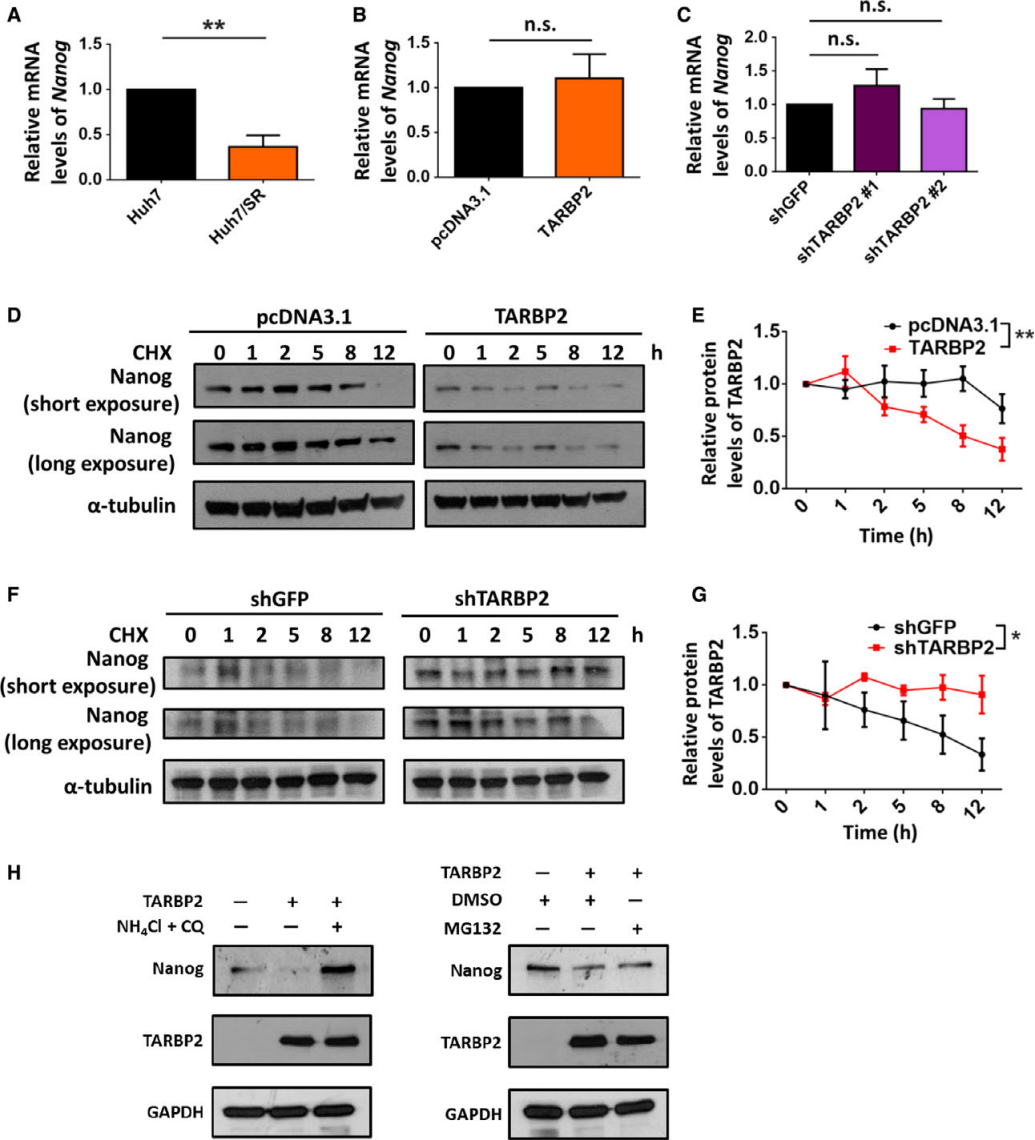
TARBP2 expression reduces Nanog protein stability. (A–C) Effect of TARBP2 on the mRNA expression of *NANOG*. CSC markers and *NANOG* mRNA expression in paired HCC cells were analyzed via real‐time PCR (A). TARBP2 was overexpressed in Huh7/SR cells for 48 h (B). TARBP2 was knocked down in Huh7 cells (C). *NANOG* mRNA expression was analyzed using real‐time PCR. Multigroup comparisons were analyzed by one‐way ANOVA with Tukey's *post hoc* test. (D–G) Effect of TARBP2 expression on Nanog protein stability. TARBP2 was overexpressed in Huh7/SR for 48 h. The cells were treated with CHX (100 μg·mL^−1^) for the indicated time periods (D). TARBP2 was knocked down in Huh7 cells. The cells were treated with CHX (100 μg·mL^−1^) for the indicated time periods (F). The relative quantity of the depicted proteins was analyzed through three independent experiments (E, G). (H) Effects of the proteolytic pathways on TARBP2‐mediated Nanog downregulation in Huh7 cells. TARBP2 was overexpressed in Huh7 cells for 24 h. The cells were treated with NH_4_Cl (10 mm) and CQ (200 μm) for 48 h or with MG132 (5 μm) for 24 h. Data are presented as mean ± SEM. Data are presented as the mean ± SEM from at least three independent experiments. Multigroup comparisons were analyzed by two‐way ANOVA with Tukey's *post hoc* test. *P* values < 0.05 were considered statistically significant. *P <* *0.05; **P <* *0.01.

**Figure 7:**
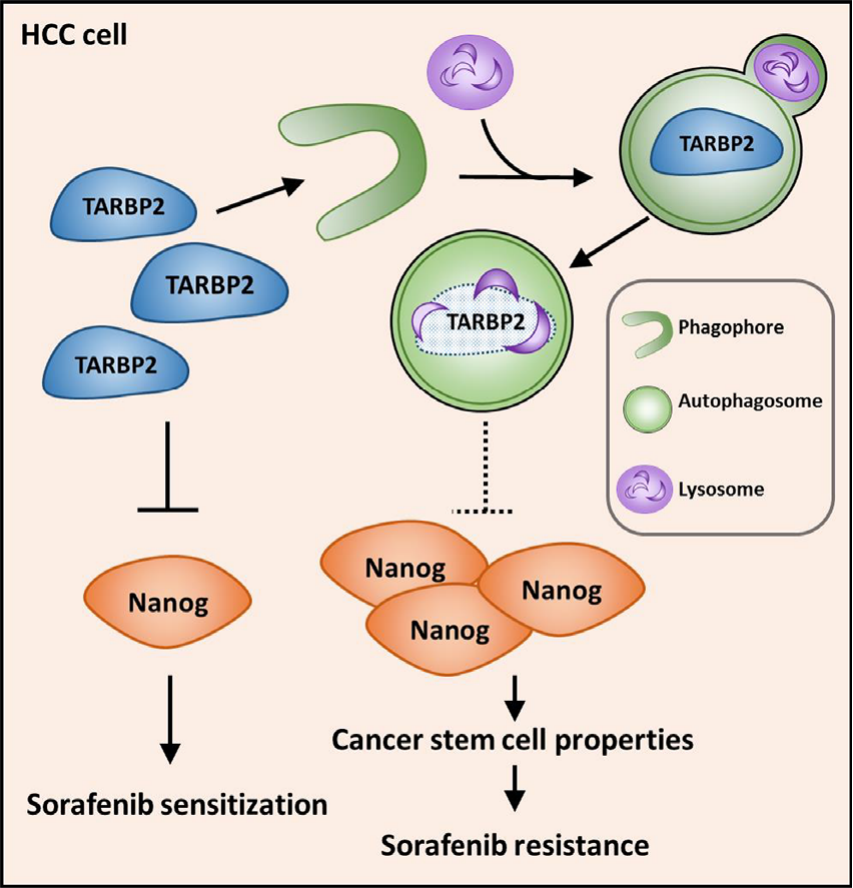
Schematic model of TARBP2‐mediated Nanog suppression in SR in HCC. TARBP2 was destabilized in HCC/SR cells through the autophagic–lysosomal proteolytic pathway. Downregulation of TARBP2 stabilized Nanog protein expression and increased CSC properties to enhance SR in HCC cells.

Because TARBP2 complements sorafenib treatment by suppressing Nanog expression in HCC cells, we next confirmed the mechanism underlying Nanog downregulation. The transcription level of *NANOG* was determined in the paired Huh7 cell lines. However, the mRNA expression of *NANOG* was decreased in the Huh7/SR cells (Fig. 6A). Owing to the contrast in Nanog protein and mRNA expression between Huh7 and Huh7/SR cells, we further verified whether *NANOG* mRNA levels were affected by this regulation and determined that *NANOG* mRNA expression was unaffected in TARBP2‐overexpressing Huh7/SR cells and TARBP2‐knockdown Huh7 cells (Fig. 6B,C). Despite the contrasting expression of Nanog protein and mRNA between Huh7 and Huh7/SR cells, further evidence demonstrated a transcription‐independent role of TARBP2 in mediating Nanog downregulation. Based on these results, we determined the protein stability of Nanog in the paired Huh7 cell lines with changes in TARBP2 expression (Fig. S2A,B). The degradation rate of the Nanog protein was enhanced in TARBP2‐overexpressing Huh/SR cells (Fig. 6D,E). Accordingly, knockdown of TARBP2 reduced the degradation rate of the Nanog protein (Fig. 6F,G), demonstrating that the stabilization of TARBP2 expression accelerates Nanog protein degradation. We further investigated whether TARBP2‐mediated Nanog protein degradation occurs through the proteasome pathway in Huh7 cells. NH_4_Cl and CQ were added to TARBP2‐overexpressing Huh7 cells to inhibit lysosome activity. Blocking the lysosome degradation pathway restored TARBP2‐mediated Nanog downregulation. Nevertheless, inhibition of proteasome‐mediated protein degradation could not prevent TARBP2‐mediated Nanog degradation, suggesting that the degradation of the TARBP2 protein in HCC/SR cells is lysosome‐dependent (Fig. 6H). Taken together, our results indicated that TARBP2 enhances sorafenib sensitization through acceleration of Nanog protein degradation in an miRNA‐independent manner. In SR HCC cells, TARBP2 is degraded through an autophagic–lysosomal pathway. Consequently, downregulation of TARBP2 enhances sorafenib resistance through stabilization of the Nanog protein in HCC cells and is correlated with poor clinical outcomes in HCC patients (Fig. 7).

## 4 Discussion

Transactivation response element RNA‐binding protein 2 has been associated with CSC features. In the present study, we identified a novel role of TARBP2 in sorafenib resistance in HCC. The TARBP2 protein was destabilized in HCC/SR cells through autophagic–lysosomal proteolytic degradation. Downregulation of TARBP2 stabilized Nanog protein expression and enhanced sorafenib resistance in HCC cells. Among the components of miRNA biogenesis, downregulation of DGCR8, Dicer, p68, and p72 has been associated with a poor prognosis in HCC (Kitagawa *et al*., 2013). Our results demonstrated that suppression of TARBP2 expression promoted sorafenib resistance in HCC cells (Fig. 2E–H), and downregulation of TARBP2 was correlated with poor outcomes in patients with HCC (Fig. 1A–D), suggesting that miRNA biogenesis factors are globally repressed in HCC cancer progression. Additionally, TARBP2 expression is reduced in several cancers, including colorectal cancer, gastric cancer, and Ewing sarcoma (De Vito *et al*., 2012; Garre *et al*., 2010; Yu and Li, 2016). However, TARBP2 functions as an oncogene to contribute to malignant transformation and proliferation in cutaneous malignant melanoma and adrenocortical carcinoma, and its expression is associated with an unfavorable prognosis in patients with breast cancer (Caramuta *et al*., 2013; Lin *et al*., 2014; Sand *et al*., 2012). These reports suggest that the effects of alteration of TARBP2 expression on cancer development are tissue‐specific.

Nanog is a pivotal transcription factor involved in the self‐renewal of CSCs and maintenance of the undifferentiated state of pluripotent cells (Gawlik‐Rzemieniewska and Bednarek, 2016; Pan and Thomson, 2007). Upregulation of Nanog has been reported to contribute to oncogenesis in multiple types of cancer (Chiou *et al*., 2010; Lin *et al*., 2012b; Meng *et al*., 2010). Notably, although a high level of Nanog expression correlates with a poor prognosis and sorafenib resistance in HCC, how Nanog is induced through these regulatory mechanisms is unknown (Jeter *et al*., 2015; Shan *et al*., 2012). We observed that downregulation of TARBP2 enhances Nanog protein expression through stabilization of the Nanog protein to render HCC cells resistant to sorafenib (Fig. 5E,F). TARBP2‐mediated Nanog protein degradation occurs via the lysosome pathway (Fig. 6). High activation of PI3K/Akt has been reported to induce sorafenib resistance in HCC (Jeter *et al*., 2015; Shan *et al*., 2012), and this activation is sustained to promote OCT4 and Nanog expression for chemoresistance and EMT in cancer cells (Almozyan *et al*., 2017), suggesting that PI3K/Akt‐mediated phosphorylation stabilizes Nanog protein expression to facilitate sorafenib resistance. The prolyl isomerase Pin1 has been shown to interact with phosphorylated Nanog. This interaction prevents the proteolysis of Nanog through inhibiting its ubiquitination. Thus, stabilized Nanog promotes self‐renewal to maintain the pluripotency of ES cells (Moretto‐Zita *et al*., 2010). In HCC, the status of Erk phosphorylation in peripheral blood mononuclear cells has been indicated as a predictor of the efficacy of sorafenib plus octreotide LAR treatment outcomes in HCC patients. In HCC patients with resistance to sorafenib plus octreotide LAR, Erk activity was observed to gradually increase after 10 days from the beginning of treatment (Caraglia *et al*., 2011). This finding is suggested that hyperphosphorylated Nanog is stabilized to confer self‐renewal and CSC features in HCC for sorafenib resistance.

Cytosolic LC3 conversion (LC3‐I to LC3‐II) functions in autophagosome formation and cargo selection and is regarded as a marker of autophagosome biogenesis (Tanida and Waguri, 2010; Tanida *et al*., 2005). TARBP2 was shown to be downregulated through an autophagic–lysosomal pathway, and we observed that the HCC/SR cells exhibited greater autophagosome formation than the parental cells (Fig. 4C,E,G), suggesting that autophagy activity is increased in Huh7/SR cells to enhance TARBP2 protein degradation. Selective autophagy is the initial process in autophagy, in which specific cellular material or organelles tagged with ubiquitin are selectively recognized (Stolz *et al*., 2014). Upregulation of miR‐423‐5p secretion in sorafenib‐treated patients with HCC has been found to result in favorable progress in relief and stabilization of the disease. The increased miR‐423‐5p inhibits the cell cycle and promotes autophagy activity (Stiuso *et al*., 2015). The miRNA biogenesis factors Dicer and Ago2 are degraded by autophagy through recognition of the autophagy receptor to promote cancer progression (Gibbings *et al*., 2012; Lai *et al*., 2018). Both Dicer and Ago2 are ubiquitylated through an E3 ligase to specifically interact with the autophagy receptor. As a component of RISC with Dicer and Ago2, TARBP2 might be degraded in cancer cells via selective autophagy. It has been suggested that sorafenib treatment promotes autophagy to selectively degrade TARBP2 and that this effect may persist, leading to sorafenib resistance (Stiuso *et al*., 2015). However, the E3 ligase of TARBP2 and ubiquitination levels need to be determined for further investigation in cancer. Supporting this concept, the SUMOylation of TARBP2 stabilizes TARBP2 protein expression through reducing its ubiquitination to suppress tumor progression, indicating that the ubiquitination of TARBP2 is essential for cancer progression (Chen *et al*., 2015). Additionally, autophagy facilitates sorafenib resistance in HCC cells (Liu *et al*., 2013; Zhai *et al*., 2014), suggesting that inhibition of autophagy resensitizes HCC cells to sorafenib treatment through blocking the degradation of TARBP2. Thus, our study may offer a potential strategy for overcoming sorafenib resistance through inhibition of autophagy activity.

Overexpression of TARBP2 induces tumor formation via inhibition of PKR phosphorylation and PKR‐mediated eIF2α phosphorylation (Benkirane *et al*., 1997; Kim *et al*., 2014). As an RNA‐binding protein, TARBP2 enhances invasion and metastatic colonization by directly binding APP and ZNF395 transcripts, thereby post‐transcriptionally enhancing their decay rate in breast cancer (Goodarzi *et al*., 2014). This evidence proves that TARBP2 exhibits an miRNA‐independent role in regulating cancer progression. By disrupting the interaction between Dicer and TARBP2, TARBP2 retains the ability to downregulate Nanog expression (Fig. 5C). Thus, the present study reveals another miRNA‐independent role of TARBP2 that destabilizes the Nanog protein and consequently resensitizes HCC cells to sorafenib treatment.

## 5 Conclusions

Transactivation response element RNA‐binding protein 2 is significantly downregulated in SR HCC cells. Restoration of TARBP2 expression resensitizes HCC/SR cells to sorafenib treatment. The TARBP2 protein is destabilized through autophagic–lysosomal proteolysis and thereby stabilizes the protein expression of the CSC marker Nanog to facilitate sorafenib resistance of HCC cells. TARBP2 expression inversely correlates with Nanog levels in SR HCC tumors. In present study, we reveal a novel miRNA‐independent role of TARBP2 in regulating sorafenib resistance in HCC cells.

## Author contributions

HHL, CWL, DLO, and PSC conceived and designed all experiments. HHL conducted the experiments on TARBP2 ΔC4. DLO performed the animal experiment. CWL performed all other experiments in this study. CCH, DLO, HYS, CFC, and PSC discussed the data. HHL, CWL, DLO, CFC, HYS, and PSC wrote and revise the paper.

## Conflicts of interest

The authors declare no conflict of interest.

## Supporting information


**Fig. S1**. TARBP2 inhibits CSCs marker expression.
**Fig. S2**. Manipulation of TARBP2 expression for analysis of Nanog protein stability.
