# Supplementary material for: TARBP2‐mediated destabilization of Nanog overcomes sorafenib resistance in hepatocellular carcinoma
Source: Mol Oncol. 2019 Feb 22;13(4):928–45. doi: 10.1002/1878-0261.12449 (PMC6441883; doi:10.1002/1878-0261.12449)
Supplement: Supplementary file 1 — Fig. S1. TARBP2 inhibits CSCs marker expression. Fig. S2. Manipulation of TARBP2 expression for analysis of Nanog protein stability. [file MOL2-13-928-s001.pdf]

Supplementary Figure S1

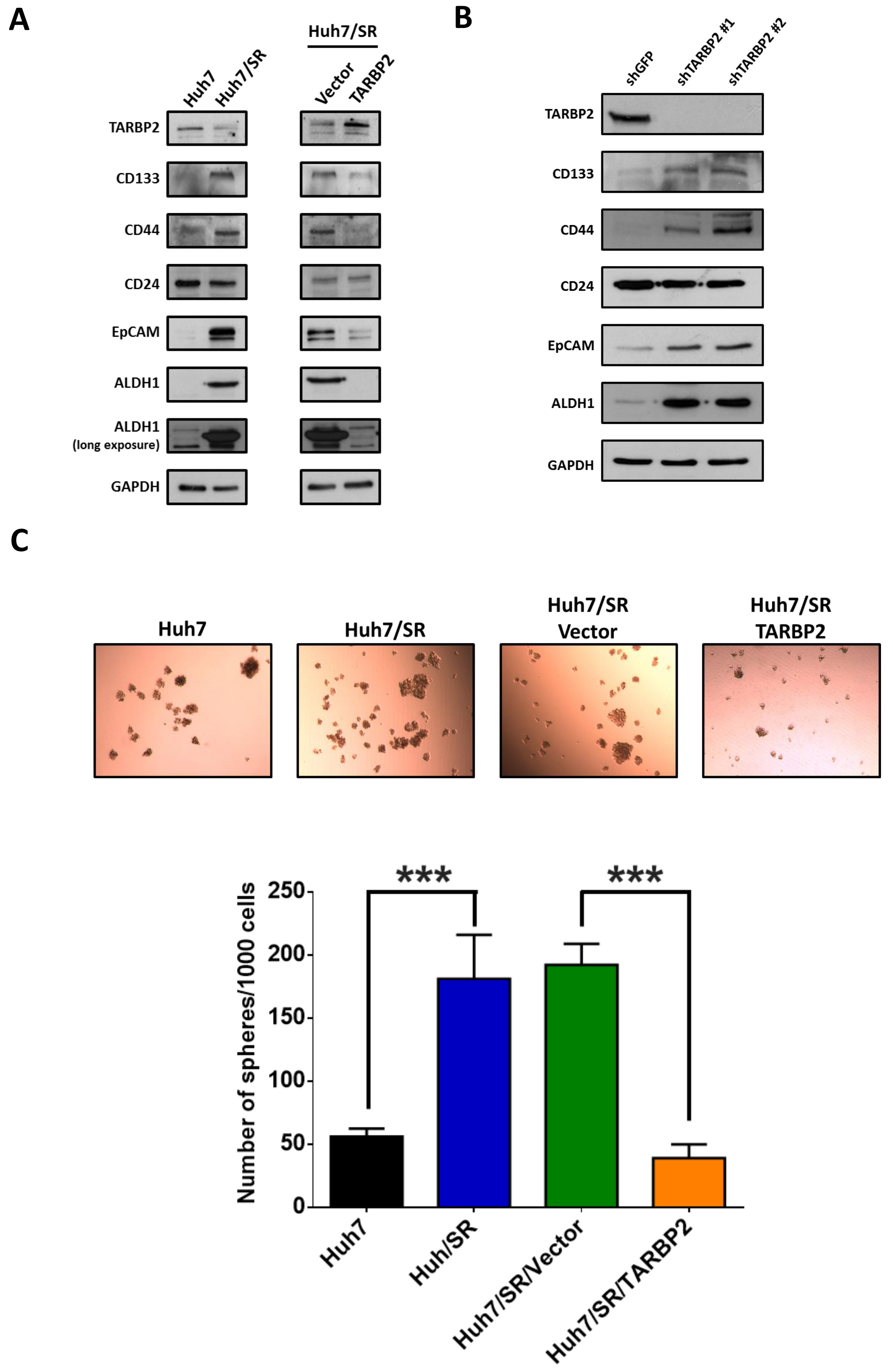

D

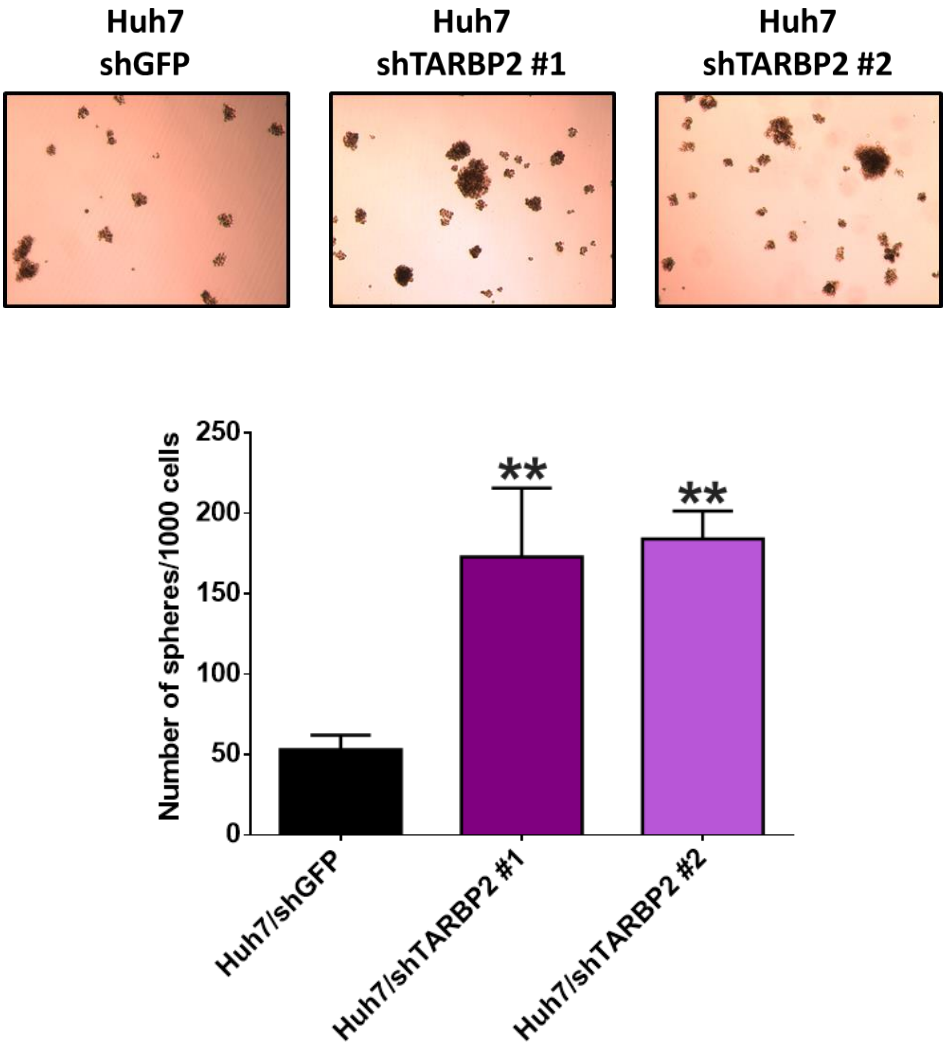

Supplementary Figure 1. TARBP2 inhibits cancer stem cell marker expression. (**A**) The protein lysates of paired Huh7 cells were harvested for western blotting (**A**, left). TARBP2 was overexpressed in Huh7/SR cells for 48 hours (**A**, right). The cancer stem cell markers CD24, CD44, CD133, EpCAM and ALDH1 were analyzed by western blot analysis. (**B**) TARBP2 was knocked down in Huh7 cells. The cancer stem cell markers were analyzed by western blot analysis. (**C**) Effects of TARBP2 on sphere formation. The paired Huh7 cells and TARBP2-overexpressing Huh7/SR cells were seeded in nonadherent plates for 14 days for sphere formation. (**D**) TARBP2 was knocked down in Huh7 cells, which were then seeded in nonadherent plates for 14 days for sphere formation. Quantification of sphere formation was performed by directly counting the number of spheres per well in plates under a 20× objective. Data are presented as mean ± SEM. Multigroup comparisons were analyzed by 1-way ANOVA with Tukey's post hoc test. P values of less than 0.05 were considered statistically significant. *$P < 0.05$; **$P < 0.01$; or ***$P < 0.001$.

Supplementary Figure S2

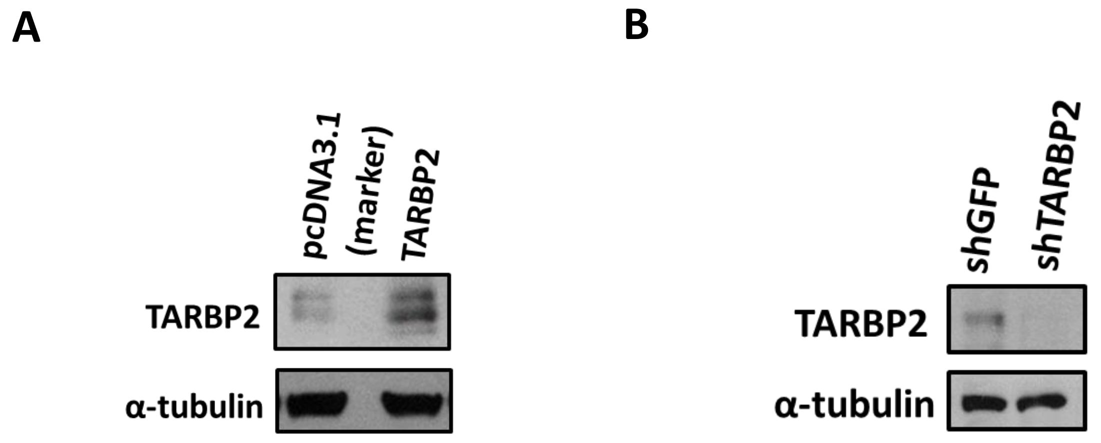

Supplementary Figure 2. Manipulation of TARBP2 expression for analysis of Nanog protein stability.

(**A** and **B**) TARBP2 was overexpressed in Huh7/SR cells for 48 hours (**A**). TARBP2 was knocked down in Huh7 cells (**B**). TARBP2 protein expression at 0 hour of CHX treatment was analyzed by western blotting.
